# Near-Complete Genome Sequence of Lötschberg Virus (*Mononegavirales*: *Filoviridae*) Identified in European Perch (Perca fluviatilis Linnaeus, 1758)

**DOI:** 10.1128/mra.00028-23

**Published:** 2023-03-16

**Authors:** Torsten Seuberlich, Jens H. Kuhn, Heike Schmidt-Posthaus

**Affiliations:** a Division of Neurological Sciences, Vetsuisse Faculty, University of Bern, Bern, Switzerland; b Integrated Research Facility at Fort Detrick, National Institute of Allergy and Infectious Diseases, National Institutes of Health, Fort Detrick, Frederick, Maryland, USA; c Institute for Fish and Wildlife Health, Vetsuisse Faculty, University of Bern, Bern, Switzerland; Katholieke Universiteit Leuven

## Abstract

We obtained the near-complete genome sequence of a novel virus, Lötschberg virus (LTBV), from a European perch metatranscriptome. Genome organization and pairwise sequence comparison indicated that LTBV represents a tentative new species and genus of the mononegaviral family *Filoviridae.*

## ANNOUNCEMENT

We previously reported the assembly of near-complete genomes of three novel viruses using metatranscriptomic data, derived from pooled organ samples of farmed European perch (Perca fluviatilis Linnaeus, 1758): Fiwi virus (FIWIV), Kander virus (KNDV), and Oberland virus (OBLV) (1). In 2022, these viruses were officially classified by the International Committee on Taxonomy of Viruses (ICTV) as members of the mononegaviral family *Filoviridae* in novel species Thamnovirus percae, Thamnovirus kanderense, and Oblavirus percae, respectively (2). Also, we found evidence of an additional potentially new virus, represented by four contigs [573 to 3,529 nucleotides (nt) in length]. The predicted encoded protein sequences were 40 to 47% identical to those of Huángjiāo virus (HUJV), a filovirid identified in greenfin horse-faced filefish [Thamnaconus septentrionalis (Günther, 1874)] (1). In that study, we performed high-throughput sequencing (HTS) in paired-end mode (2 × 150 nt), but reads were unpaired for sequence assembly due to limited computational memory (300 GB RAM) (1). Using upgraded computational memory (2 TB RAM), we reassembled the HTS reads in paired-end mode, which increased the assembly efficiency and resulted in a single near-complete genomic sequence of this virus, which we named Lötschberg virus (LTBV).

Clinically sick European perch were obtained from an aquaculture farm in Switzerland (1). We extracted RNA from pooled organ samples (brain, spleen, kidneys, heart, and pyloric ceca) with TRI reagent (Sigma Life Sciences). HTS libraries were prepared with the TruSeq stranded total RNA kit (Illumina). We sequenced the libraries in paired-end mode, with 150 cycles at a sequencing depth of 2 × 10^9^ on a HiSeq 3000 system (Illumina). Reads were trimmed using fastp (v. 0.12.5; parameters -l 33 -W 4 -M 15 -5 3 -3 3) (3). Host-derived sequences were removed by aligning reads to the European perch genome (GENO_Pfluv_1.0) (4), using STAR (v. 2.7.3a) (5). Nonaligned reads were assembled with SPAdes (v. 3.12.0) (6). The resulting scaffolds were screened for homologs against the National Center for Biotechnology Information (NCBI) nonredundant protein sequence database using the blastx command in Basic Local Alignment Search Tool (BLAST) and DIAMOND (v. 2.0.9) (7), with a default *e*-value cutoff of 0.001. The sequences were manually annotated in Geneious Prime (v. 2023.0.1).

**FIG 1:**
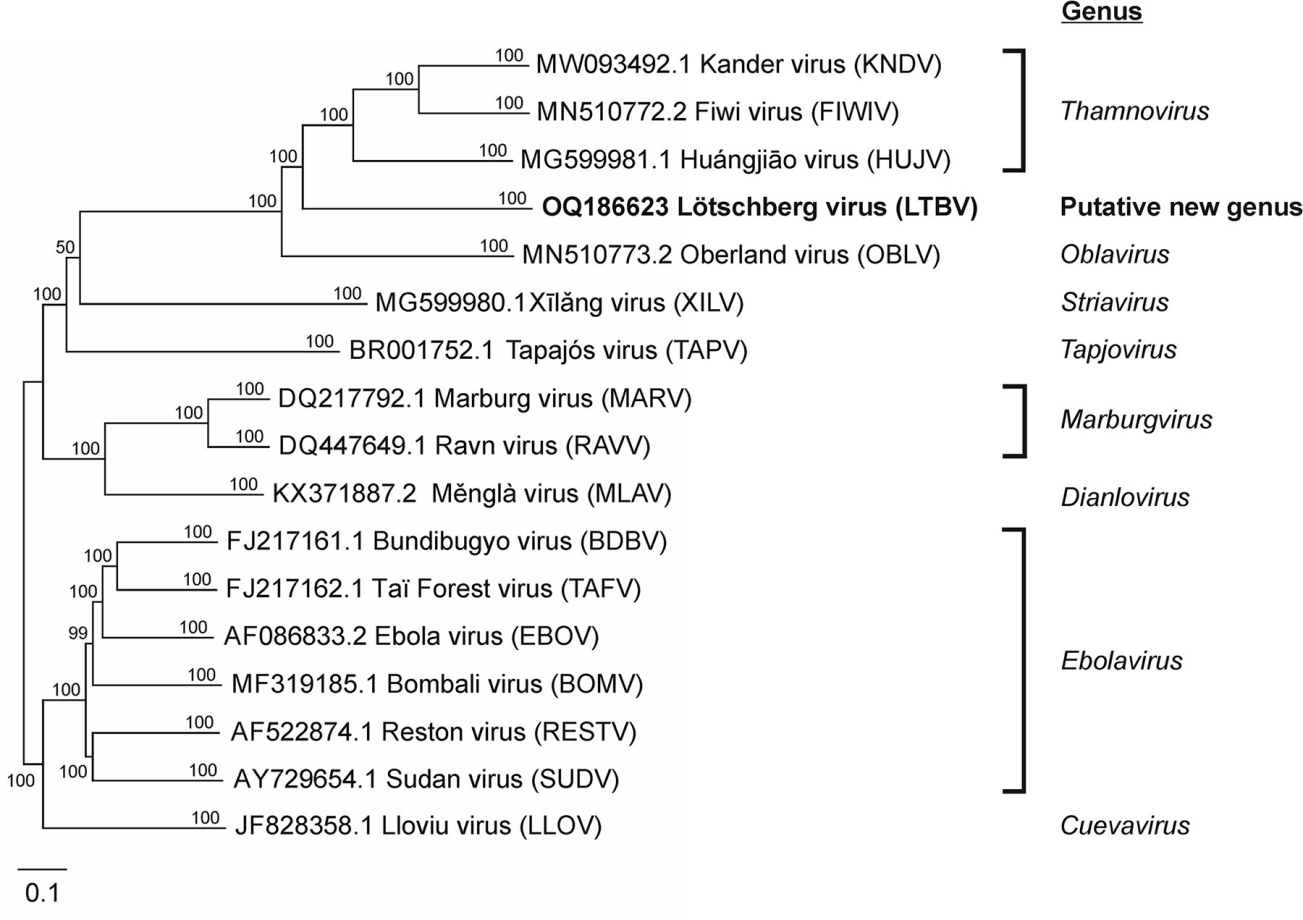
Phylogenetic position of Lötschberg virus (LTBV) in the mononegaviral family *Filoviridae*. The neighbor-joining tree was built based on near-complete genome sequences after sequence alignment with Clustal Omega (v. 1.2.2, default settings) in Geneious Prime (Dotmatics, v. 2023.0.1) and inferred with the Geneious Consensus Tree Builder setup (Jukes-Cantor model; 5,000 bootstraps). GenBank accession numbers are indicated for each sequence at branch tips.

We obtained a scaffold of 13,584 nt (all bases assigned, no ambiguities) with a GC content of 47.7% and an average depth coverage of 12.8×. The sequence contained seven open reading frames (ORFs) (>70 codons), organized similarly to those of filovirid oblaviruses and thamnoviruses, encoding filovirid-typical proteins: nucleoprotein (NP), polymerase cofactor (VP35), glycoprotein (GP_1,2_), transcriptional activator (VP30), and large protein (L)—plus two new proteins of unknown function. The 3′ and 5′ termini and the terminal ORFs remained incomplete (Table 1). Comparison of LTBV NP, VP35, GP_1,2_, VP30, and L amino- acid sequences with the homologous FIWIV, HUJV, KNDV, and OBLV proteins revealed 23 to 48% sequence identity. Pairwise Sequence Comparison (PASC; https://www.ncbi.nlm.nih.gov/sutils/pasc/viridty.cgi?textpage=overview) of near-complete genome sequences indicates that LTBV is most closely related to thamnoviruses (up to 40% PASC similarity), which is also supported by phylogenetic analysis (Fig. 1). Based on the current demarcation criteria for filovirid species (≥23% PASC divergence) and genera (≥55% PASC divergence) (8, 9), LTBV represents a member of a tentative new species and genus within the family *Filoviridae.*

**TABLE 1:** Comparison of the reported genomic sequence lengths and the length of the open reading frames (ORFs) of Lötschberg virus with those of related fish filovirids

Virus	Reported genomic sequence length (nt)	Open reading frame (ORF) length (nt)						
		ORF 1	ORF 2 (NP)	ORF 3 (VP35)	ORF 4	ORF 5 (GP)	ORF 6 (VP30)	ORF 7 (L)
Lötschberg virus	13,584^*a*^	336^*b*^	1,200	1,680	294	1,800	1,206	6,215^*b*^
Oberland virus	14,682^*a*^	466^*b*^	1,200	1,968	957	1,263	1,152	6,656^*b*^
Kander virus	13,849^*a*^	654^*b*^	1,206	1,665	279	1,791	1,374	6,373^*b*^
Fiwi virus	13,764	468	1,203	1,662	276	1,860	1,077	6,435
Huángjiāo virus	14,280	666	1,215	1,686	273	1,881	1,185	6,441

a Near-complete genome sequence.

b Incomplete open reading frame.

### Data availability.

HTS raw data have been deposited in the NCBI Sequence Read Archive (SRA) under accession no. SRR12586223 (https://www.ncbi.nlm.nih.gov/sra/?term=SRR12586223). The genome sequence of LTBV is available at GenBank under accession no. OQ186623 (https://www.ncbi.nlm.nih.gov/nuccore/?term=OQ186623).
